# Clinical Features, Management, and Prognostic Factors of Intracranial Solitary Fibrous Tumor

**DOI:** 10.3389/fonc.2022.915273

**Published:** 2022-05-30

**Authors:** Jingdian Liu, Sisi Wu, Kai Zhao, Junwen Wang, Kai Shu, Ting Lei

**Affiliations:** Department of Neurosurgery, Tongji Hospital, Tongji Medical College, Huazhong University of Science and Technology, Wuhan, China

**Keywords:** intracranial solitary fibrous tumor, clinical outcome, prognostic factors, radiotheapy, surgery treatment

## Abstract

**Background:**

Because of the low incidence and the constantly changing diagnostic and classification criteria, the clinical features, management, and prognostic factors of intracranial solitary fibrous tumor (ISFT) remain unclear and were thus analyzed in this study.

**Method:**

A total of 38 patients with ISFTs who were diagnosed in our institution were enrolled in this study. Patient data including age, gender, clinical presentation, histopathological features, immunohistochemistry staining, tumor location, tumor size, treatment methods, and prognosis were extracted and retrospectively analyzed.

**Results:**

The median age at diagnosis was 45.5 years (range 28–66 years) and the male-to-female ratio was 1:1.53 in our series. The 3-, 5-, and 10-year progression-free survival (PFS) rate was 82.2%, 62.8%, and 21.4%, respectively; and the 3-, 5-, and 10-year overall survival rate was 97.1%, 86.9%, and 64.2%, respectively. Patients with high WHO grade (grade 3) ISFTs experienced impaired PFS (p < 0.05) and OS (p < 0.01). Subtotal resection (STR) was associated with worse PFS and OS (p < 0.001, respectively). Postoperative radiotherapy (PORT) improved PFS, especially local control rate, in patients with WHO grade 3 ISFTs (P = 0.025) or STR (p = 0.027). Moreover, CD34-negative immunostaining and a high Ki-67 index (>10%) were associated with impaired PFS in ISFTs.

**Conclusion:**

Our study provides evidence that high tumor grade, subtotal tumor resection, CD34 negative immunostaining, and high Ki-67 index (>10%) were independent predictors for the poor prognosis of ISFTs. PORT can improve local control rate, and should be recommended for patients with high-grade ISFTs or STR.

## Introduction

Solitary fibrous tumor (SFT) was first described as a rare mesenchymal neoplasm arising from pleura by Klemperer and Rabin in 1931 (1). Although this type of tumor has been identified in nearly every anatomic site of the body, the intracranial origin is less common. Intracranial SFT (ISFT) comprises less than 1% of all primary brain tumors (2). Originally, ISFTs and hemangiopericytomas (HPC) were regarded as two separate neoplasms due to their distinct biological behaviors. With the development of sequencing technologies, the NGFI-A–binding protein 2 signal transducer and activator of transcription 6 (NAB2-STAT6) fusion gene was detected in both SFT and HPC (3). The special NAB2-STAT6 fusion protein can drive tumor growth by activating the EGR gene (3). The discovery resulted in the combination of these two diseases into a single entity, and the combined term “solitary fibrous tumor/hemangiopericytoma” was proposed in the 2016 World Health Organization (WHO) classification of central nervous system (CNS) tumors (4). According to the latest version in 2021, the term “hemangiopericytoma” has been removed to conform fully with soft tissue pathology nomenclature, with the tumor now termed only “SFT” (5).

ISFTs are difficult to distinguish radiologically from meningiomas because of their overlapping imaging features (6). Traditionally, some immunohistochemistry markers (for example, CD34 and CD99) were employed for the diagnosis of ISFTs. However, this can be problematic because these markers can be also detected in other brain tumors (7). After the discovery of NAB2-STAT6 fusion gene, accumulating evidence has found that STAT6 nuclear staining is extremely sensitive and specific in ISFTs, which made STAT6 immunohistochemistry a powerful diagnostic modality (8).

Because of the low incidence and the changes in WHO classification and diagnostic criteria over the years, the knowledge of natural course and prognostic factors of ISFTs is still limited. Hitherto, little information about ISFTs has been reported in the literature. Most previous reports either exhibited small patient series or confused HPCs with SFTs as the same tumors (9–11). In this present study, we included 38 patients with ISFTs and analyzed their clinical characteristics and follow-up outcomes to gain novel insight into the management of this disease.

## Materials and Methods

From March 2008 to September 2020, 38 patients with primary ISFT underwent surgical treatments in the neurosurgery department of Tongji Hospital, Huazhong University of Science and Technology. Patients with any other cancers or severe chronic basic diseases were excluded. Patient information including age at the time of surgery, gender, clinical manifestation, histopathologic features, tumor location, imaging features, treatment methods, survival status, and survival time were retrospectively collected. This study was approved by local ethical authorities in accordance with the Helsinki Criteria. Written informed consent was obtained from each individual patient or from family members of those who had died.

The extent of tumor resection was determined by surgical notes and postoperative neuro-imaging findings. Gross total resection (GTR) was equivalent to Simpson grades I and II, whereas others were considered subtotal resection (STR). The selection of adjuvant radiotherapy depended on the patient’s willingness, extent of surgery resection, WHO grade, and clinical presentation. Progression-free survival (PFS) was determined as the time interval from the date of surgery to the time of tumor progression or recurrence, which was identified by follow-up magnetic resonance imaging (MRI) after surgery for patients. Tumor recurrence was classified as local, regional, or distant. Local recurrence referred to an event within 2 cm (the maximum margin for the clinical target volume (CTV) from gross tumor volume in patients with PORT) from surgical area. Regional recurrence was defined as a remote intracranial recurrence beyond primary tumor site. Distant recurrence meant extracranial metastasis of the tumor. Survival time was calculated from the date of the surgery to the time of death. Surviving patients were censored at the time of last follow-up. The first postoperative MRI was performed 1 month after surgery, and then, the follow-up interval was extended to 3 months. Survival data of patients were obtained through outpatient and telephone follow-up.

The diagnosis was confirmed by neuropathology experts through postoperative genetic and histopathologic examination of tumor samples. In addition, three most common primer pairs were designed and employed to identify the NAB2-STAT6 fusion subtypes by reverse transcriptase polymerase chain reaction (RT-PCR) in 33 samples (12) (**Supplementary Table S1**). Five other tissues were excluded due to poor tissue preservation. As described previously, immunohistochemistry staining was performed to detect expression of STAT6, CD34, S-100, Ki-67, vimentin (VIM), epithelial membrane antigen (EMA), and glial fibrillary acidic protein (GFAP) (13).

Quantitative variables and categorical variables were compared by Student’s t-test and chi-square test (or Fisher’s exact test), respectively. The effects of each factor on PFS and OS were evaluated by Kaplan–Meier method and univariate/multivariate cox regression method. A p-value < 0.05 was considered significantly different. R software (version 4.0.2) was used for performing all statistical analysis and graphing.

## Results

The clinical and histopathological characteristics of 38 patients with ISFTs were summarized in **Table 1**. The median age of patients at the first surgery after diagnosis was 45.5 years (range, 28–66 years). Fifteen patients were male (39.5%), and 23 were female (60.5%) with a male-to-female ratio of 1:1.53. The majority of the tumors (63.2%) were supratentorial, whereas 14 were located in the infratentorial region. The average size (maximum diameter) of tumors was 5.1 cm. The most common symptom was headache which occurred in 27 patients (71.1%). Other symptoms included epilepsy (n = 5, 13.2%), limb weakness (n = 7, 18.4%), visual impairment (n = 5, 13.2%), and paresthesia (n = 3, 7.9%). Two patients (5.3%) were asymptomatic, and the tumors were detected by routine clinical examinations. According to postoperative histopathological findings, nine (23.7%) patients were diagnosed with WHO grade 3, 17 (44.7%) with WHO grade 2, and 12 (31.6%) with WHO grade 1.

**Table 1:** Characteristics of 38 patients.

Characteristics		Numbers (%)
**Age (years)**		
20–40		11 (28.9%)
41–60		24 (63.2%)
>61		3 (7.9%)
Median		45.5
Range		28–66
**Gender**		
Male		15 (39.5%)
Female		23 (60.5%)
**Location of tumor**		
Supratentorial		24 (63.2%)
Infratentorial		14 (36.8%)
**Size**		
<5 cm		17 (44.7%)
≥5 cm		21 (55.3%)
Mean		5.1 ± 2.2
**Extent of surgery**		
GTR		29 (76.3%)
STR		9 (23.7%)
**Radiotherapy**		
Yes	GTR (range, 55–62 Gy; 1.8–2.3 Gy/fractionation)	6 (15.8%)
	STR (range, 53–65 Gy; 1.8–2.2 Gy/fractionation)	5 (13.2%)
	Total	11 (28.9%)
No		27 (71.1%)
**WHO grade**		
1		12 (31.6%)
2		17 (44.7%)
3		9 (23.7%)
**Ki-67 index**		
1-5%		21 (55.3%)
6-10%		9 (23.7%)
>10%		8 (21.1%)
**CD34**		
Positive		28 (73.7%)
Weak positive		5 (13.2%)
Negative		5 (13.2%)
**Recurrence**		
Local		13 (34.2%)
Regional		3 (7.9%)
Distant		2 (5.3%)

**Figure 1:**
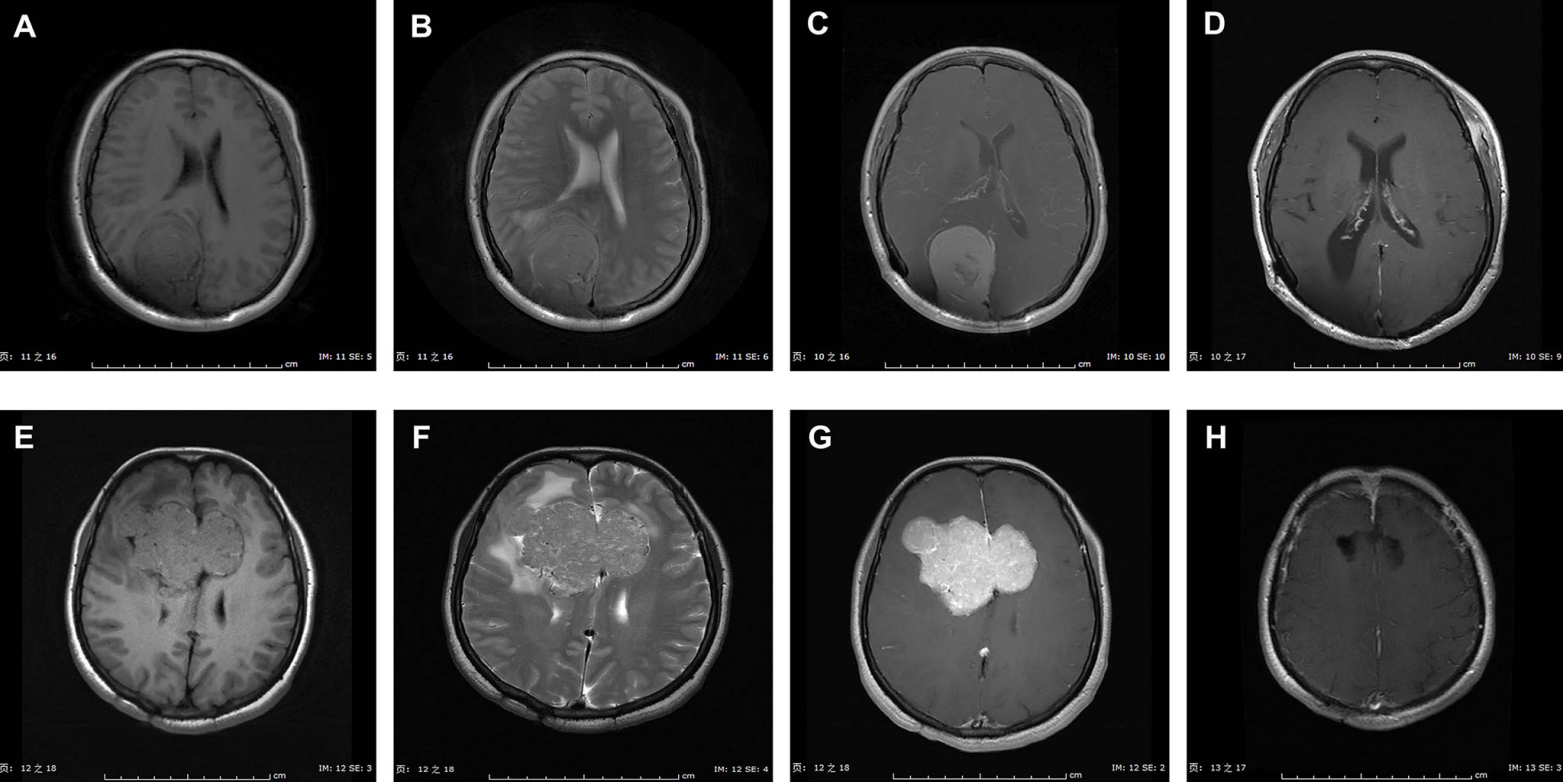
Representative MRI scans of ISFTs in our series. Generally, the signal intensity of the tumor mass was heterogeneous mixed isointense and hypointense on non-contrast T1 and T2 MRI sequences, and marked and heterogeneous enhancement on T1 with gadolinium contrast (T1-Gd) MRI scan. **(A–D)** MRI scans of a 47-year-old female with ISFT lesion located in right occipital lobe. **(A–C)** Preoperative axial T1, T2, and T1-Gd MRI scans. **(D)** Postoperative T1-Gd MRI scan showed a GTR resection. **(E–H)** MRI scans of a 44-year-old female with ISFT mass originated from callosum and invaded into both sides of frontal lobe. **(E–G)** Preoperative axial T1, T2, and T1-Gd MRI scans. **(H)** Postoperative T1-Gd MRI scan demonstrated a GTR resection.

**Figure 1** exhibited the representative MRI images of ISFTs. Generally, the signal intensity of the tumor mass was heterogeneous mixed isointense and hypointense on non-contrast T1 and T2 MRI sequences, and marked and heterogeneous enhancement on T1 with gadolinium contrast (T1-Gd) MRI scan.

**Figure 2:**
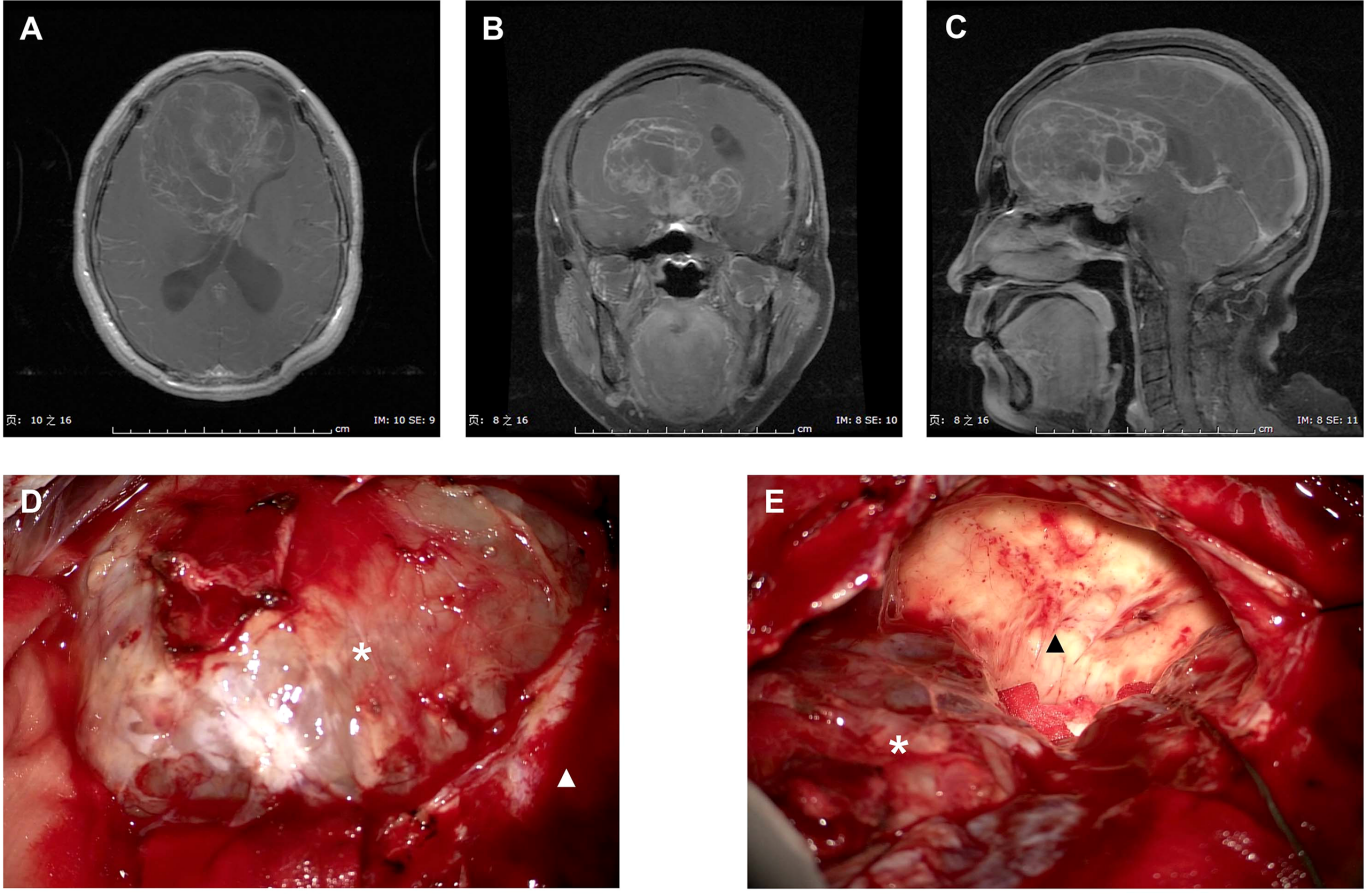
Intraoperative images for tumor resection of a recurrent ISFT. **(A–C)** Preoperative axial, coronal, and sagittal T1-Gd MRI scans of a huge recurrent ISFT tumor mainly located in the right frontal skull base, showing a marked and heterogeneous enhancement. **(D, E)** Intraoperative images revealed a red solid tumor mass (black arrow), which was not clearly distinguishable from the normal brain tissue during tumor resection (white arrow). Finally, a subtotal resection was performed due to the tumor invasion of skull base bone and sellar construction with a serious intraoperative bleeding (about 1,000 ml).

**Figure 2** presented the intraoperative images for tumor resection of a recurrent ISFT. A STR was finally performed in this case due to the tumor invasion of skull base bone and sellar construction with a serious intraoperative bleeding (about 1,000 ml).

All 38 patients underwent long-term follow-up after the first surgery, and the mean follow-up period was 66.6 months (range, 12–165 months). Five patients experienced postoperative complications: three patients got cerebrospinal fluid leakage, one patient had incision infection, and one patient developed hydrocephalus, which was subsequently cured by ventriculo-peritoneal shunt. Eighteen cases suffered from recurrence during follow-up period, and seven patients elapsed. Recurrence was determined as local in 13 patients, regional in three patients (one patient with grade 3 and two patients with grade 2), and distant in two patients (two patients with grade 3). The 3-, 5-, and 10-year PFS rate was 82.2%, 62.8%, and 21.4%, respectively. Overall survival (OS) rate was 97.1% for 3 years, 86.9% for 5 years, and 64.2% for 10 years.

**Figure 3:**
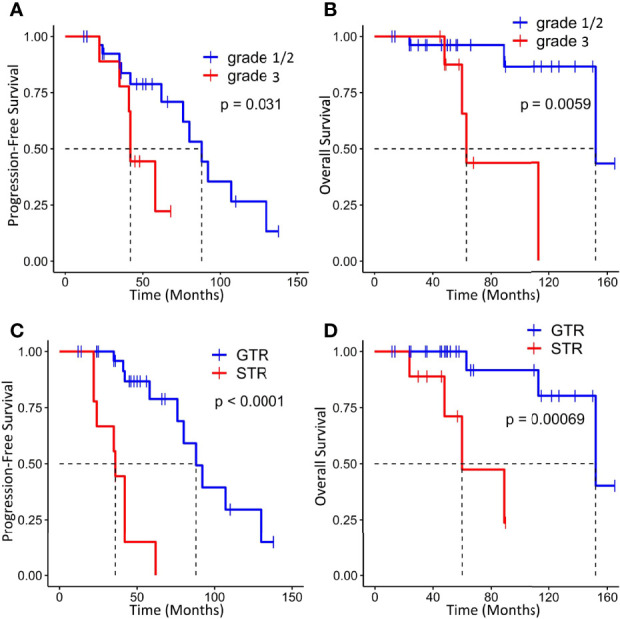
Kaplan–Meier survival curves for progression-free survival and overall survival based on WHO grade **(A, B)** and extent of resection **(C, D)**.

On the basis of the Kaplan–Meier survival analysis, patients with WHO high-grade ISFTs had a lower PFS (median PFS 42 vs. 88 months, p = 0.031) and OS (median OS 63 vs. 152 months, p = 0.0059) compared with lower-grade counterparts (**Figures 3A, B**). Moreover, a markedly decreased recurrence or progression rate and prolonged survival were observed in patients with GTR (median PFS 88 vs. 36 months, p < 0.0001; median OS 152 vs. 60 months, p = 0.00069) (**Figures 3C, D**). In addition, univariate/multivariate Cox regression analysis was performed to further verify this result, as shown in **Table 2**. Patient age, gender, tumor location (infratentorial or supratentorial), and tumor size were not correlated with prognosis (**Table 2**).

**Table 2:** Results of univariate and multivariate cox regression analysis. .

	PFS-Univariate Analysis			PFS-Multivariate Analysis		
Factors	HR	95%CI	P-value	HR	95%CI	P-value
Age	1.00	0.95–1.05	0.960	–	–	–
Gender (female vs. male)	1.05	0.40–2.80	0.916	–	–	–
Location (Infratentorial vs. Supratentorial)	1.08	0.41–2.85	0.876	–	–	–
Size (≥5 cm *vs*. <5 cm)	0.47	0.17–1.29	0.134	–	–	–
WHO grade (3 vs. 1/2)	3.36	1.07–10.6	0.044*	4.24	1.29–13.91	0.017*
The extent of surgery (STR vs. GTR)	11.94	3.50–40.85	<0.001*	13.55	3.90–47.03	<0.001*
Radiotherapy (Yes vs. No)	1.57	0.60–4.15	0.368	–	–	
	**OS-Univariate analysis**			**OS-Multivariate analysis**		
**Factors**	**HR**	**95%CI**	**P-value**	**HR**	**95%CI**	**P-value**
Age	1.02	0.93–1.10	0.703	–	–	–
Gender (female vs. male)	1.16	0.20–6.47	0.866	–	–	–
Location (Infratentorial vs. Supratentorial)	1.80	0.36–8.98	0.479	–	–	–
Size (≥5 cm vs. <5 cm)	0.82	0.18–3.69	0.796	–	–	–
WHO grade (3 vs. 1/2)	8.38	1.44–48.7	0.014*	18.71	1.81–193.4	0.014*
The extent of surgery (STR vs. GTR)	16.75	1.85–151.4	0.004*	35.52	2.447–515.6	0.009*
Radiotherapy (Yes vs. No)	1.56	0.34–7.12	0.570	–	–	–

P* values are statistically significant. HR, hazard ratio; CI, confidence interval.

**Figure 4:**
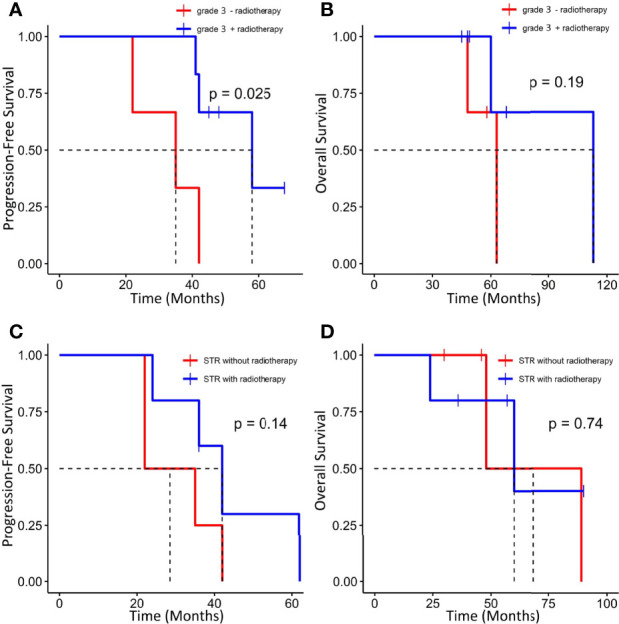
Kaplan–Meier survival curves for progression-free survival and overall survival based on PORT. **(A, B)** Effect of PORT on PFS and OS in patients with WHO grade 3 ISFTs. **(C, D)** Effect of PORT on PFS and OS in patients with STR.

**Figure 5:**
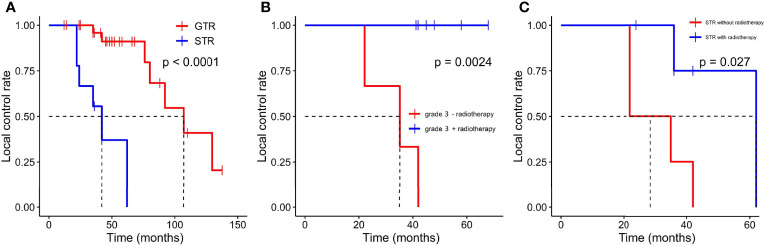
Kaplan–Meier survival curves for local control rate. **(A)** The effect of resection extension on local control rate. **(B)** The effect of PORT on local control rate in patients with WHO grade 3 ISFTs. **(C)** the effect of PORT on local control rate in patients with STR.

GTR was achieved in 29 patients (76.3%), whereas others underwent STR due to illegible boundary between tumor mass and surrounding normal tissues. In our series, none of the cases received chemotherapy. Postoperative radiotherapy (PORT) (11 patients were treated) was performed predominately in patients with high-grade tumors or STR (range, 53–65 Gy; 1.8–2.3 Gy/fractionation). The median margin for the CTV) was 10 mm (range, 5–20 mm) from gross tumor volume (GTV). Five of nine patients treated with STR received PORT, whereas six of nine diagnosed with WHO grade 3 ISFTs received PORT, and three patients refused due to personal financial reasons or opposition of their families. The effect of radiotherapy on prognosis is not significant when the analysis was performed across all patients (**Table 2**), but in patients with WHO grade 3 ISFTs, PORT remarkably suppressed the progression/recurrence of tumors but did not improve OS (median PFS: 58 vs. 35 months, p = 0.025; median OS: 63 vs. 113 months, p = 0.19) (**Figures 4A, B**). In addition, an improvement of PFS could be observed in patients treated with PORT in the STR subgroup (median PFS: 42 vs. 28.5 months, p = 0.14) (**Figure 4C**); however, no statistical differences were established, owing to the small case series. The impact of PORT on OS of patients with STR was not observed (Median OS: 60 vs. 68.5 months, p = 0.74) (**Figure 4D**). Furthermore, we also analyzed the impact of surgical extent and PORT on different patterns of recurrence (**Table 3**). Local recurrence was the main progression type (26.1% after GTR and 100% after STR, respectively) in the subgroup of patients without radiotherapy. According to the results of Kaplan–Meier analysis, GTR significantly decreased the local recurrence (**Figure 5A**). Moreover, PORT significantly suppressed the local recurrence in patients with WHO grade 3 ISFTs or in those with STR (**Figures 5B, C**).

**Table 3:** Patterns of recurrence according to the type of treatment.

		n	Local	Regional	Distant
**GTR**	**PORT (+)**	6	1 (16.7%)	1 (16.7%)	1 (16.7%)
	**PORT (−)**	23	6 (26.1%)	1 (4.3%)	0
**STR**	**PORT (+)**	5	2 (40%)	1 (20%)	1 (20%)
	**PORT (−)**	4	4 (100%)	0	0
**p-value**			0.026*	0.340	0.149

Chi-square test (or Fisher’s exact test). *P < 0.05.

According to the results of IHC staining, the positive rate of STAT6, VIM, S-100, EMA, and GFAP was 92.1%, 94.7%, 39.4%, 23.7%, and 5.3%, respectively. The level of expression of CD34 was classified as follows: diffuse positive (28 cases, 73.7%), weakly (focal) positive (five cases, 13.2%), and negative (five cases, 13.2%). The mean value of Ki-67 index was 8.5% (range, 1%–30%). The CD34 expression and WHO grade showed a significant negative correlation (p = 0.011) (**Table 4**). Moreover, high-grade ISFTs had a higher Ki-67 index compared with low-grade tumors (average value: 13.4% for grade 3 vs. 8.2% for grade 2 vs. 5.3% for grade 1, p = 0.034).

**Table 4:** Correlation analysis between IHC markers and WHO grade.

	WHO 1/2	WHO 3	P-value
**STAT6**			–
Positive	26	9	
Negative	3	0	
**VIM**			0.423
Positive	28	8	
Negative	1	1	
**S-100**			0.273
Positive	13	2	
Negative	16	7	
**EMA**			–
Positive	9	0	
Negative	20	9	
**GFAP**			–
Positive	2	0	
Negative	27	9	
**CD34**			0.011*
Positive	23	5	
Weakly positive	5	0	
Negative	1	4	

P* values are statistically significant.

The NAB2-STAT6 fusion subtypes were detected and summarized in **Table 5**. NAB2ex4–STAT6ex2, NAB2ex6–STAT6ex16, and NAB2ex6–STAT6ex17 fusion variants were detected in 5, 12, and 9 cases, respectively. Seven cases could not be distinguished by the aforementioned NAB2–STAT6 fusion. Notably, three STAT6-negative ISFTs were confirmed to harbor NAB2ex6–STAT6ex16 fusion. No statistical differences were established for the prognosis between variable fusion subtypes in this study.

**Table 5:** NAB2-STAT6 fusion subtypes in 33 patients with ISFTs.

NAB2-STAT6 Fusion Type	N	%
EX4-EX2	5	15.2%
EX6-EX17	9	27.3%
EX6-EX16	12	36.4%
Not applicable	7	21.2%
Total	33	100%

**Figure 6:**
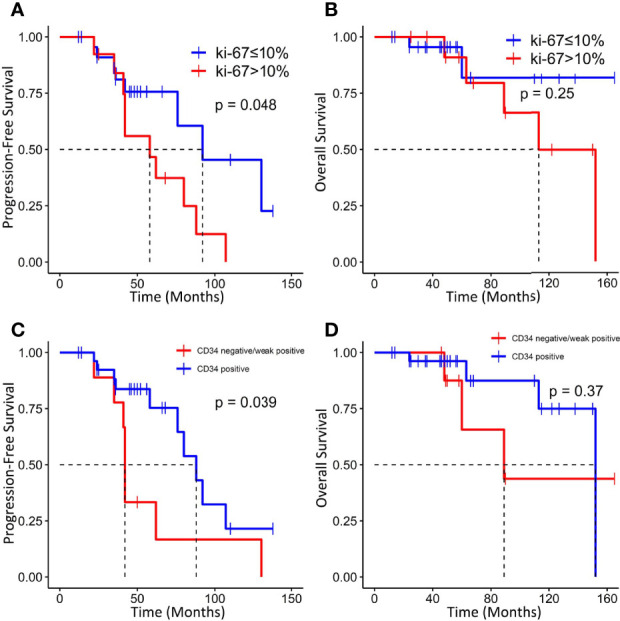
Kaplan–Meier survival curves for progression-free survival and overall survival based on Ki-67 index **(A, B)** and CD34 **(C, D)**.

Tumors with a higher Ki-67 index (>10%) were associated with worse PFS (median PFS: 58 vs. 92 months, p = 0.0164) but not OS (**Figures 6A, B**). In addition, absent/low expression of CD34 in ISFTs portended an unfavorable prognosis (median PFS: 42 vs. 88 months, p = 0.039; median OS: 89 vs. 152 months, p = 0.37) (**Figures 6C, D**).

## Discussion

Although SFTs and HPCs have been consolidated into a single disease according to the 2013 (fourth edition) and 2020 (fifth edition) WHO classification of tumors of soft tissue and bone, the term “hemangiopericytoma” is still used widely by neuropathologists (14). Traditionally, ISFTs were considered to be benign with a low possibility of relapse and an indolent course, whereas HPCs exhibited a local aggressive behavior (15). However, some SFTs with malignant features were continuously reported (16). Thus, the misdiagnosis between SFTs and HPCs occurs frequently during the past years. The distinction between the two types was no longer clinically significant due to the pronounced clinical and histopathological overlap. In the 2021 WHO classification of CNS tumors, the term “hemangiopericytoma” was removed with the tumor named only “SFT” (5). Because of the constantly changing classification criteria in recent years, the clinical features and management guidelines of ISFTs remain unclear. To fill this gap, we conducted this retrospective study by analyzing the clinical and follow-up data of 38 patients with ISFTs.

Unlike other types of brain tumors, patients with ISFTs usually have a younger onset age, with a peak in the 40–60 years (7). The patients aged 41–60 years in our department occupied up to nearly 64%. Early perspectives thought that ISFTs were more predominant in men, whereas the opposite trend was observed in this study (17). In addition, approximately equal gender distribution was recently reported by a population-based study (18). The symptoms of patients with ISFTs are non-specific in our cohort, which is aligned with other studies (9). Headache is the most common presentation in brain tumors due to the tissues compression or increased intracranial pressure caused by tumor growth. Other neurologic symptoms including epilepsy, limb weakness, paresthesia, and visual impairment are associated with tumor location and nerves invasion. In addition, previous studies reported some uncommon symptoms covering anosmia, memory loss, dysphasia, hyponatremia, amenorrhea, and hypoglycemia (9).

In the majority of previous studies, WHO grade and the extent of resection are commonly reported as prognostic factors of ISFTs, which is in line with ours (10, 11). Multivariate analysis indicated that GTR remarkably prolonged OS and PFS of patients, regardless of tumor grade and other confounders. For the vast majority of benign ISFTs, GTR is independently sufficient to achieve clinical cure. Notwithstanding, in our cohort, two of 10 patients with WHO grade 1 ISFT receiving GTR still developed local recurrences at 9 and 11 years after surgery, respectively. Moreover, a recent study reported that longer recurrence intervals along with malignant transformation occurred in some WHO grade 1 ISFTs (19). For this reason, long-term follow-up should be warranted irrespective of the grade of ISFTs. In terms of higher grade ISFTs, frequent recurrence occurred after GTR due to higher mitotic activity and with microscopic residual disease (18).

Many researchers reported that patients could benefit from radiotherapy after surgery (18, 20). However, the benefit may be specific to patients with STR or high grade ISFTs and be highly confined to local control. In our series, the effect of radiotherapy on PFS and OS was not significant when the analysis was performed across all patients (**Table 2**). In STR subgroup, the PFS of patients with STR + PORT was improved compared with those who received STR alone, although the effect was also not significant (**Figure 4C**). Taking into consideration that the patients without PORT had a higher local recurrence rate (**Table 3**), we analyzed the effect of PORT on local control rate. Interestingly, PORT significantly improved the local control rate in patients with STR (**Figure 5C**). It is odd that the patients receiving PORT had much higher regional and distant recurrence rates in this cohort. This might be because the majority (55%) of patients with PORT were diagnosed as WHO grade 3 ISFTs, which generally had more malignant phenotypes.

Little literature reported the effect of radiotherapy on GTR subgroup. A recent study reported that patients with GTR can benefit from PORT (20). Unfortunately, this issue was difficult to be evaluated in our series, because of the small case series and the selection bias that the vast majority of people with RT in GTR subgroup were the patients with WHO grade 3 ISFTs. Recently, two based-population studies found that GTR with radiation significantly improved disease-free survival compared with GTR alone in borderline malignant or malignant ISFTs (18, 21). Our results also suggested that PORT can improve PFS and local control rate for patients who were diagnosed with WHO grade 3 tumors. In contrast, the clinical value of PORT in benign ISFTs is still debatable. Moritani et al. reported that progression and dedifferentiation of an ISFT were probably related to the application of radiation therapy (22). In addition, in five patients with lower-grade ISFTs in which malignant transformation occurred, two cases underwent RT after initial surgery (19). However, the association between radiotherapy and malignant transformation of benign ISFTs needs further confirmation. Altogether, PORT should be recommended primarily for patients with high grade ISFTs or those with STR. For the patients with benign ISFTs who underwent GTR, the role of PORT requires further evaluation in the future.

Shin et al. assessed the significance of preoperative radiotherapy in ISFTs (23). They observed a worse RFS and OS in patients who received radiotherapy before resection though further validation would be required. On the other hand, it is also difficult to selectively implement preoperative radiotherapy only for the patients with high-grade tumors, because of the plight of identification of tumor histological type and tumor grade before resection.

IHC staining characterized by mainly STAT6 is the key to the diagnosis of ISFTs. Although ISFTs were confused with meningiomas on imaging, the STAT6 immunostaining is totally negative in meningiomas (7). However, absence of STAT6 nuclear expression by IHC staining may not exclude the possibility of SFT. The sensitivity of STAT6 for ISFTs was reported to be 96.6% by analysis of a literature review, resembling a 92.1% positive rate in the present study (7). For diagnosis of STAT6-negative ISFTs, combination with other IHC markers is helpful, although their specificities for ISFTs are not so high as STAT6 (24). In addition, molecular diagnostic techniques such as RT-PCR could be helpful for STAT6-negative SFTs by detecting the NAB2–STAT6 fusion (12, 25). As a transmembrane glycoprotein, CD34 was identified in hematopoietic stem and progenitor cells, fibroblast-related mesenchymal cells, and endothelial cells (26). Before the discovery of STAT6-NAB2 fusion gene, positive expression of CD34 was regarded as the most prominent characteristic of ISFTs and was often used for differential diagnosis (27). However, 5%–10% of SFTs were negative for CD34 (7, 28). A study found that the absence of CD34 may be related to dedifferentiation of SFT (29). In addition, a recent clinicopathologic study of 25 cases with loss of CD34 reported that CD34-negative SFTs are more likely to exhibit malignant behaviors, compared with their CD34-positive counterparts (28). Although the authors reported the correlation between prognosis of patients with SFTs and CD34, the majority of the reported cases are extracranial and the significance of CD34 in ISFTs is still unclear (28). As indicated in the results section, we found that reduced/absent expression of CD34 was associated with degree of malignancy of ISFT and tumor progression. Similarly, Yamashita et al. described that expression level of CD34 gradually decreased with increased malignancy of tumors in 163 patients with ISFTs (7). A low expression rate (<10%) of CD34 also was reported in 60% of recurrent cases by Bertero and his colleagues (10). Moreover, some studies found that loss of CD34^+^-fibrocytes was frequently observed in other types of invasive carcinoma (e.g., invasive lobular carcinoma of the breast and invasive cervical carcinoma) (30, 31). As one type of antigen-presenting cells, loss of CD34^+^-fibrocytes may promote immune evasion of tumor (32). A high Ki-67 (a proliferation marker) index was also identified as a risk factor for tumor recurrence in our study and others (33).

In conclusion, we assessed the clinical feature and prognosis of 38 patients with ISFTs in this study. The results suggested that tumor grade and extent of surgery resection are independent prognostic factors of ISFTs. PORT could improve PFS, especially decreased local recurrence for patients with high grade ISFTs or those with STR. Moreover, CD34-negative ISFT or a high Ki-67 index might be a prediction of poor prognosis.

## Data Availability Statement

The original contributions presented in the study are included in the article/**Supplementary Material**. Further inquiries can be directed to the corresponding authors.

## Ethics Statement

The studies involving human participants were reviewed and approved by the ethics committee of Tongji Hospital, Tongji Medical College. The patients/participants provided their written informed consent to participate in this study.

## Author Contributions

JL and SW contributed equally in this work. KS and TL designed and conducted this project. JL, SW, KZ, and JW collected the data and run statistical analysis. JL and SW wrote the original manuscript. KS and JW revised the manuscript. All authors contributed to the article and approved the submitted version.

## Funding

Natural Science Foundation of Hubei Province (2020CFB657).

## Conflict of Interest

The authors declare that the research was conducted in the absence of any commercial or financial relationships that could be construed as a potential conflict of interest.

## Publisher’s Note

All claims expressed in this article are solely those of the authors and do not necessarily represent those of their affiliated organizations, or those of the publisher, the editors and the reviewers. Any product that may be evaluated in this article, or claim that may be made by its manufacturer, is not guaranteed or endorsed by the publisher.
