# Supplementary material for: Clinical Features, Management, and Prognostic Factors of Intracranial Solitary Fibrous Tumor
Source: Front Oncol. 2022 May 30;12:915273. doi: 10.3389/fonc.2022.915273 (PMC9197442; doi:10.3389/fonc.2022.915273)
Supplement: Supplementary file 1 [file Table_1.docx]

Supplementary Table S1：List of the Primers Used.

| Type | Sequence | |
| --- | --- | --- |
| EX4-EX2 | F | CCCGAGAGAGCACCTACTTG |
|  | R | GGTGCTGGACAGTGTCTGAA |
| EX6-EX17 | F | ACATCCTGCAGCAGACACTG |
|  | R | TCTGGGGTAGGAAGTGGTTG |
| EX6-EX16 | F | AGCAGACACTGATGGACGAG |
|  | R | TGGGCTTCTTGGGATAGAGA |
